# Autoantibody profiles and clinical association in Thai patients with autoimmune retinopathy

**DOI:** 10.1038/s41598-021-94377-0

**Published:** 2021-07-22

**Authors:** Aulia Rahmi Pawestri, Niracha Arjkongharn, Ragkit Suvannaboon, Aekkachai Tuekprakhon, Vichien Srimuninnimit, Suthipol Udompunthurak, La-ongsri Atchaneeyasakul, Ajchara Koolvisoot, Adisak Trinavarat

**Affiliations:** 1 Faculty of Medicine, Universitas Brawijaya, Malang, Indonesia; 2 Department of Ophthalmology, Faculty of Medicine Siriraj Hospital, Mahidol University, 2 Wanglang Road, Bangkok Noi, Bangkok, 10700 Thailand; 3 Research Division, Faculty of Medicine Siriraj Hospital, Mahidol University, Bangkok, Thailand; 4 Nuffield Department of Medicine, Welcome Center for Human Genetics, University of Oxford, Oxford, UK; 5 Division of Medical Oncology, Department of Medicine, Faculty of Medicine Siriraj Hospital, Mahidol University, Bangkok, Thailand; 6 Clinical Epidemiology Division, Siriraj Medical Research Center, Faculty of Medicine Siriraj Hospital, Mahidol University, Bangkok, Thailand; 7 Division of Rheumatology, Department of Medicine, Faculty of Medicine Siriraj Hospital, Mahidol University, 2 Wanglang Road, Bangkok Noi, Bangkok, 10700 Thailand

**Keywords:** Immunology, Autoimmunity, Diagnostic markers

## Abstract

Autoimmune retinopathy (AIR) is a rare immune-mediated inflammation of the retina. The autoantibodies against retinal proteins and glycolytic enzymes were reported to be involved in the pathogenesis. This retrospective cohort study assessed the antiretinal autoantibody profiles and their association with clinical outcomes of AIR patients in Thailand. We included 44 patients, 75% were females, with the overall median age of onset of 48 (17–74, IQR 40–55.5) years. Common clinical presentations were nyctalopia (65.9%), blurred vision (52.3%), constricted visual field (43.2%), and nonrecordable electroretinography (65.9%). Underlying malignancy and autoimmune diseases were found in 2 and 12 female patients, respectively. We found 41 autoantibodies, with anti-α-enolase (65.9%) showing the highest prevalence, followed by anti-CAII (43.2%), anti-aldolase (40.9%), and anti-GAPDH (36.4%). Anti-aldolase was associated with male gender (*P* = 0.012, OR 7.11, 95% CI 1.54–32.91). Anti-CAII showed significant association with age of onset (*P* = 0.025, 95% CI − 17.28 to − 1.24), while anti-α-enolase (*P* = 0.002, OR 4.37, 95% CI 1.83–10.37) and anti-GAPDH (*P* = 0.001, OR 1.87, 95% CI 1.32–2.64) were significantly associated with nonrecordable electroretinography. Association between the antibody profiles and clinical outcomes may be used to direct and adjust the treatment plans and provide insights in the pathogenesis of AIR.

## Introduction

Autoimmune retinopathy (AIR) is a rare immune-mediated inflammation of the retina that can eventually lead to total blindness. The clinical outcomes are diverse, some of which result from cone dysfunctions, such as reduction in visual acuity and abnormal color vision, or rod-associated conditions, including peripheral vision loss, ring scotomas, and nyctalopia^1^. This condition is marked by the late onset, usually in the fifth or sixth decade of life, sudden progression of bilateral retinal degeneration, and associated autoimmune diseases in the patient or their family members. Generally, AIR is classified into two groups: the non-paraneoplastic autoimmune retinopathy (npAIR) and paraneoplastic autoimmune retinopathy (pAIR). The pAIR is subclassified into cancer associated retinopathy (CAR) and melanoma associated retinopathy (MAR). Circulating antiretinal autoantibodies are believed to be the cause of AIR^2^. A molecular mimicry-induced autoimmunity could provide the explanation for both npAIR and pAIR^3^. In npAIR, the mimicry might occur between the microbial antigen and retinal proteins^4^, while in pAIR, the autoantibodies are raised against the tumor neoantigens, which show high similarity to the retinal self-antigens^5^.

Several groups of autoantibodies have been revealed by probing the patient sera against retinal extract and purified retinal proteins^3,6,7^. The autoantibodies against retinal proteins, such as recoverin, arrestin, transducin, rhodopsin, tubby-like protein 1 (TULP1), carbonic anhydrase II (CAII), heat shock protein 27 (HSP27), HSP60, Rab6, and collapsin response mediator protein (CRMP), can be detected in the AIR patient sera^4,8^. Among these autoantibodies, anti-recoverin is the most specific biomarker for AIR, although it is not sufficient to be the sole biomarker^9^. The other antigen group targeted by autoantibodies in AIR are glycolytic enzymes, such as α-enolase, aldolase, glyceraldehyde-3-phosphate dehydrogenase (GAPDH), and pyruvate kinase (PK). Autoantibodies to these antigens might result from response to microbial infection, tumor, other autoimmune diseases, or possibly released from damaged cells^2,10^. Although they can act as potential biomarkers, the presence of autoantibodies towards these ubiquitous proteins is non-specific, since they often overlap in other clinical features^5,11^. Moreover, not all AIR patients have detectable titers of antiretinal autoantibodies in immunoblot^12^. These uncertainties put the diagnosis of AIR using antiretinal autoantibodies detection still not a definitive verdict.

The correct diagnosis of AIR is essential, since the treatment using immunosuppressants differ from those of other retinal dystrophies or degenerations^13^. Due to the unavailability of a standard testing method, AIR remains the diagnosis of exclusion by combining the clinical manifestation, the absence of other possible etiologies, and the presence of antiretinal autoantibodies^14,15^. The lack of internationally standardized and validated diagnostic criteria and laboratories, and the limitations surrounding the use of antiretinal autoantibodies as predictive biomarkers contribute to the underestimation of AIR prevalence^5,11^. Despite its limitations, the detection of antiretinal antibodies is still an indispensable tool for the diagnosis of AIR. Understanding the autoantibody profiles and related clinical presentation in the population is essential in establishing the sensitive diagnosis of AIR.

This study is the first to explore the antiretinal autoantibody profiles in AIR patients in Thailand. We also sought to determine the association between the autoantibodies and the clinical outcomes. Thorough evaluation of these autoantibody profiles in combination with the clinical assessment is expected to provide a robust diagnosis, leading to appropriate treatment to minimize the aggravation of the disease.

## Methods

### Study design and ethics

This retrospective cohort study assessed the secondary data of clinical presentation, history, and antiretinal autoantibody profiles from medical records of suspected AIR patients in Siriraj Hospital, Thailand. All procedures were performed in accordance with the relevant guidelines and regulations. The study was approved by the institutional review board (IRB) of the Faculty of Medicine Siriraj Hospital Mahidol University (approval number SI531/2020, dated June 24th, 2020). All the data that we analyzed and reported, including the patient history, ophthalmological examination, and antibody detection in patient sera, had been collected in the course of routine diagnostic analysis for suspected AIR patients. Therefore, the IRB had approved the exempt of informed consent from individual patients.

### Data collection

Due to the rarity of AIR, we reviewed medical records of all AIR patients attending the Ophthalmology clinic from November 2013 until December 2020. The suspicion of AIR cases began by ruling out other possible causes of visual abnormality and signs of severe inflammation. Additional data comprised clinical presentations, including decreased best corrected visual acuity (BCVA), abnormal color vision, nyctalopia, or reduced peripheral visual field (VF), personal or family history of autoimmune diseases and/or malignancies, abnormal electroretinography (ERG), optical coherence tomography (OCT), and fundus photography. Finally, the diagnosis of AIR cases was supported by the positive result of at least one antiretinal autoantibody.

### Antiretinal autoantibody detection

As part of routine diagnosis for suspected AIR cases, patient sera were collected and sent to the Ocular Immunology Laboratory, Casey Eye Institute, Oregon Health & Science University, Portland, OR, USA. The reactivity was tested using immunoblotting. The first 20 patients were tested for autoantibodies against retinal and other unidentified proteins prior to the availability of the specific diagnostic panels. Afterwards, 22 patients were subjected for the autoimmune retinopathy panel (CAII, HSP27, retinal arrestin, tubulin, GAPDH, aldolase, α-enolase, and PKM2), while two patients with history of cancer were tested for the cancer-associated retinopathy panel (recoverin, CAII, HSP60, TULP1, Rab6, GAPDH, aldolase, α-enolase, and PKM2).

### Statistical analysis

The demographic data and antibody profiles were presented descriptively. The association between categorical or ordinal variables were assessed using chi-square and Fisher exact test. The mean/median between variables with continuous data was analyzed using independent t-test or Mann–Whitney U test. The hypothesis was two-tailed with *P* < 0.05 considered as statistically significant. All missing data were excluded from analysis. The statistical analysis was performed using PASW Statistics version 18.0.0.

## Results

### Baseline characteristics of patients

There were 44 patients with suspected AIR (Table 1 and Supplementary Table 1), 33 females and 11 males, with the median age of onset and at diagnosis of 48 (17–74, IQR 40–55.5) and 58.5 (23–75, IQR 50.75–64) years, respectively. In the first visit, common clinical presentations were nyctalopia (65.9%), blurred vision (52.3%), and reduction in peripheral VF (43.2%). More than half of the patients presented with normal color vision (59.1%) and nonrecordable ERG (65.9%). Interestingly, we found 12 (all female) out of 44 patients with underlying autoimmune diseases (systemic lupus erythematosus (SLE) 50%, rheumatoid arthritis (RA) 33%, and others 17%) and two female patients with confirmed malignancy (CAR). Most patients (65.9%) reported neither related underlying conditions nor family history of autoimmune diseases. The median age of onset in patients with underlying autoimmune disease was significantly lower than those without (40 vs 50 years, *P* = 0.014) by chi-square test. Two patients with CAR had the age of onset of 48 and 50 years. The average BCVA at baseline was 0.6 logMAR, where 64.4% of evaluated eyes presented with good vision (BCVA ≤ 0.54 logMAR), 13.8% with moderate visual acuity, and 21.8% with legal blindness (≥ 1.00 logMAR). VF examination revealed that all of 79 evaluated eyes from 42 patients (two had no VF data and five had VF data only in one eye) had a constricted VF, with a mean reduction of 77.3%. Sixty-six (83.5%) of 79 evaluated eyes had a reduction of more than 50% from the normal value, 16.5% had a reduction of less than 50%. Central and paracentral scotoma were found in 3.8% and 27.8% of the evaluated eyes, respectively. Five patients (11.4%) had a history of receiving chloroquine or hydroxychloroquine for autoimmune conditions, for which data was available from four patients. The average duration of the treatment in these patients was 3.75 years. Three patients had the onset of AIR 2, 3, and 9 years after discontinuing chloroquine, while in one patient, the ophthalmological symptoms began during chloroquine treatment.

**Table 1:** Baseline characteristics of the suspected AIR patients.

Variable		Frequency (percentage)
Age of onset (years)	< 20	1/44 (2%)
	20–40	11/44 (25%)
	41–60	22/44 (50%)
	> 60	10/44 (23%)
Gender	Female	33/44 (75%)
	Male	11/44 (25%)
Color vision	Normal	26/44 (59%)
	Total color blindness	6/44 (14%)
	Partial color blindness	1/44 (2%)
	Cannot be evaluated due to low vision	9/44 (21%)
	N/A	2/44 (5%)
ERG	Reduced rod and cone responses	9/44 (21%)
	Reduced cone response, normal rod response	1/44 (2%)
	Reduced rod response, normal cone response	1/44 (2%)
	Absence of rod response, reduced cone response	1/44 (2%)
	Asymmetrical	1/44 (2%)
	Nonrecordable	29/44 (66%)
	N/A	2/44 (5%)
Clinical symptoms	Blurred vision	23/44 (52%)
	Nyctalopia	29/44 (66%)
	Reduction in peripheral visual field	19/44 (43%)
	Others^a^	5/44 (11%)
	No symptoms	2/44 (5%)
Relevant underlying condition	Autoimmune disease^b^	12/44 (27%)
	Malignancy	2/44 (5%)
	Family history of autoimmune disease	2/44 (5%)
	None	29/44 (66%)
BCVA (logMAR, eyes)	Good (< 0.54)	56/87 (64%) ^c^
	Moderate (0.54–1.00)	12/87 (14%) ^c^
	Legally blind (≥ 1.00)	19/87 (22%) ^c^
Visual field (eyes)^d^	Reduced < 50% from normal visual field	13/79 (17%)
	Reduced > 50% from normal visual field	66/79 (84%)
	Central scotoma	3/79 (4%)
	Paracentral scotoma	22/79 (28%)
	Cannot be evaluated (eyes)	8/87 (9%) ^c^
Intraocular lens (eyes)		28/87 (32%) ^c^
Previous medications	Chloroquine or hydroxychloroquine	5/44 (11%)

*BCVA* best corrected visual acuity, *N/A* not applicable.

^a^Photoaversion, photosensitivity, photopsia, hemeralopia, eye pain, dry eye.

^b^Systemic lupus erythematosus (SLE), rheumatoid arthritis (RA), pemphigus vulgaris, minimal change disease (MCD), undifferentiated connective tissue disease (UCTD).

^c^One patient had one prosthetic eye.

^d^There were two patients without VF data and five patients with VF data only in one eye.

### Follow up data

In this retrospective cohort study, we followed the patients ranging from 1 month to 12 years 3 months (median 1.8 years, IQR 1–3.9). The ophthalmological examination during the follow up was summarized in Table 2 and Supplementary Table 2 in the Supplement. The BCVA was decreased in 42.5% of evaluated eyes and remained stable in 27.6%. Interestingly, we found 29.9% of eyes with slight improvements in the BCVA. The patients with improved BCVA comprised of follow up time of less than or equal to one year (four patients) and more than one year (ten patients). We also observed the changes of the VF in 59 eyes with available follow up data, with periods between the examinations ranging from 0.7–6.8 (median 2.4, IQR 1.5–3.83) years. The peripheral VF was reduced in 55.9% of evaluated eyes, with an average reduction of 36.3% from the baseline, and stable in 15.3%. We noticed improvements of less than 50% from the baseline VF in 25.4% of eyes and more than 50% in 3.4%.

**Table 2:** Summary of patient follow up.

Variable		Frequency (percentage)
Follow up period (years)	Median 1.8 (0.08–12.25)	
BCVA (eyes)	Improved	26/87 (30%)^a^
	Stable	24/87 (28%)^a^
	Decreased	37/87 (43%)^a^
Duration between the first and last VF test (years)	Median 2.4 (0.7–6.8)	
Visual field change (eyes)	Improved	17/59 (29%)^b^
	Stable	9/59 (15%)^b^
	Reduced	33/59 (56%)^b^
	Not evaluated	28/87 (32%)^a^
Duration between the first and last OCT (years)	Median 2 (0.5–10)	
OCT	Stable	21/33 (64%)
	Worsen	11/33 (33%)
	N/A	11/44 (24%)
Immunohistochemistry	Positive	18/22 (82%)
	Negative	4/22 (18%)
	N/A	22/44 (50%)
Number of antiretinal antibodies	Mean ± SD 4 ± 2 Range 1–11	
Duration from onset to treatment (years)	Median 7 (0.25–30)	
Treatment	Prednisolone	30/44 (68%)
	Azathioprine	14/44 (32%)
	Methotrexate	8/44 (18%)
	Mycophenolate	7/44 (16%)
	Others ^c^	3/44 (7%)
	No treatment	12/44 (27%)

*BCVA* best corrected visual acuity, *OCT* optical coherence tomography, *VF* visual field, *N/A* not applicable.

^a^One patient had a prosthetic eye.

^b^VF was compared to the baseline.

^c^Cyclosporine, leflunamide, dexamethasone, intravenous immunoglobulins (IVIG), rituximab.

**Figure 1:**
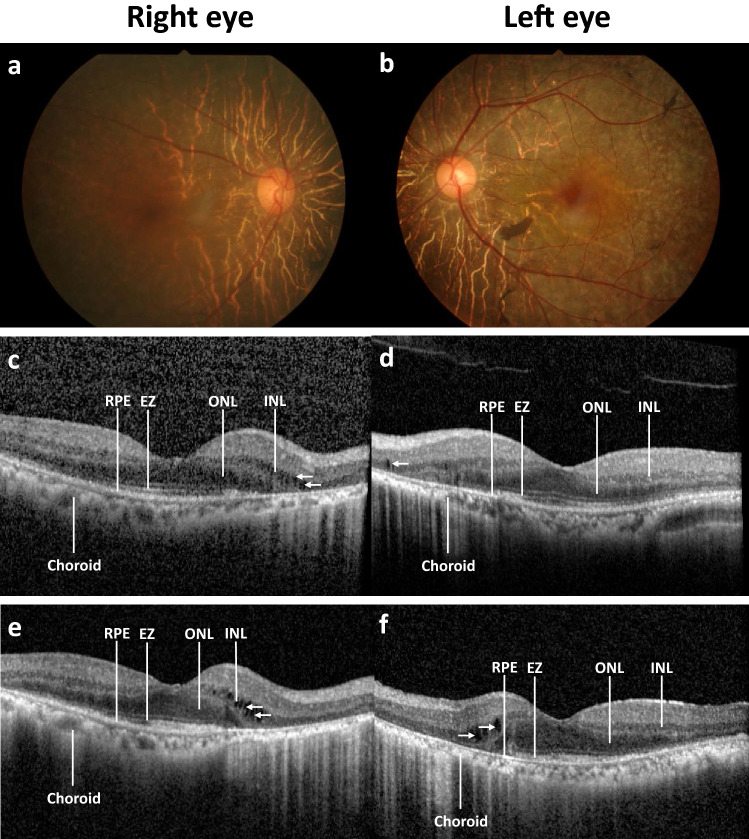
Disease progression of a patient with autoimmune retinopathy (AIR). Color fundus photography at baseline in the right (**a**) and left eye (**b**) shows normal optic disc, arteriolar attenuation, generalized retinal pigment epithelium (RPE) atrophy with macular sparing and scattered pigment clumps. Prominent large choroidal vessels can be observed around the optic disc. Optical coherence tomography images at baseline showing RPE attenuation, loss of ellipsoid zone (EZ) at the periphery, flattening of the outer nuclear layer (ONL), and slit cavitation in the inner nuclear layer (INL) on the nasal side, as marked by arrows, in the right (**c**) and left eye (**d**). The disease progression after five years is marked with RPE atrophy, progressive loss of EZ and ONL toward the fovea, more prominent slit cavitation in the INL, as marked by arrows, and accompanying choroidal thinning in the right (**e**) and left eye (**f**). The data is retrieved from patient number 1.

The baseline OCT was evaluated in 43 out of 44 patients, however the follow up data was only available from 33 patients with follow up time ranging from 0.5 to 10 (median 2, IQR 1–5) years. Compared to the baseline, 57.6% of patients showed stable OCT findings (mostly in patients with follow up time less than four years), 33.3% had worsening conditions, and 9.1% displayed no pathology. In most patients, the disease progression advanced from the peripheral retina to the macula, except in two patients, where the central retina showed more severe pathology than the peripheral areas. The overall OCT observation showed that the loss of ellipsoid zones (EZ) occurred early. The foveal EZ were usually preserved until the late stage of the disease before they eventually vanished. We found significant association between the duration of disease (the time between onset until diagnosis) and conditions of EZ at baseline (*P* = 0.007). Despite the worsening of the retinal layers and reduction of peripheral VF, some patients maintained good BCVA due to the preservation of the EZ in the fovea. Retinal pigment epithelium (RPE) changes started with attenuation, followed by atrophic disappearance. The outer nuclear layer (ONL) became thinner to flat. In patients with longer follow up periods, the choroid also showed thinning due to atrophy of the inner layer. The fundus photography appearance in most of our patients displayed generalized RPE atrophy, scattered pigment clumps, and arteriolar attenuation, as shown in Fig. 1a,b. The example of OCT from one of the patients demonstrated RPE attenuation, shortening of EZ, and flattening of the ONL (Fig. 1c–f).

Thirty-two patients (72.7%) received immunosuppressive medications, predominantly prednisolone (68.2%) and azathioprine (31.8%). The duration from the onset of the eye symptoms to the initial drug treatment ranged from 3 months to 30 years (median 7 years). We found that treatment was significantly associated with follow up variables, such as changes in VF (*P* = 0.009), OCT (*P* = 0.009), and the changes of EZ (*P* = 0.002) using chi-square test. In the group receiving treatment, 17 out of 32 (53%) patients showed stable BCVA, and 22 out of 31 (71%) patients displayed stable conditions of the EZ.

### Autoantibody profile

**Figure 2:**
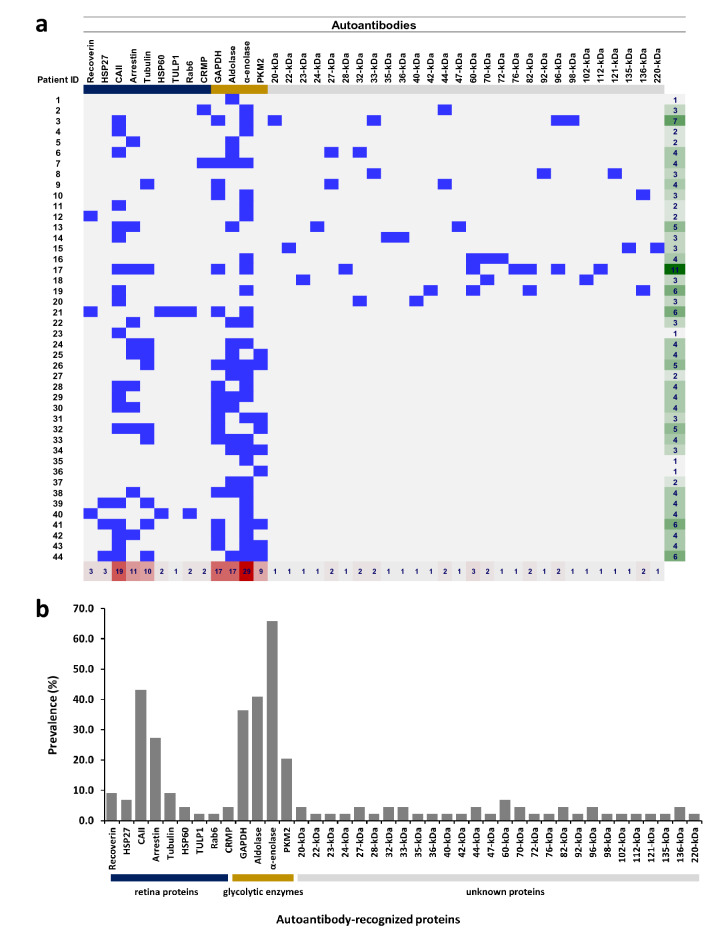
**(a)** Autoantibody profile in patients. The antibody array depicts the positive autoantibodies against the corresponding proteins (blue boxes), negative results are shown as grey boxes and white boxes indicate that the test was not assigned. The colored line under the antibody names described the retina proteins (dark blue), glycolytic enzymes (yellow), and unidentified retina proteins (grey). The last column on the right describes the total number of antibodies in each patient (green gradient) and the lowest row shows the number of patients having each type of antibody (red gradient). **(b)** Prevalence of autoantibodies in 44 patients. Bar chart depicts the prevalence of the autoantibodies based on the three protein panels: retina proteins (dark blue), glycolytic enzyme (yellow), and unidentified retina proteins (grey). *HSP* heat shock protein, *CAII* carbonic anhydrase II, *TULP1* tubby-like protein 1, *CRMP* collapsin response mediator protein, *GAPDH* glyceraldehyde 3-phosphate dehydrogenase, *PKM2* pyruvate kinase isozyme M2, *kDa* kilo Dalton.

Out of 44 patients with positive antiretinal autoantibodies, we observed 41 different autoantibodies, with average number of antibodies found in each patient 4 ± 2 (mean ± SD, range 1–11). In the present study, the autoantibodies were classified into three groups based on their target proteins: antibody against glycolytic enzymes, retinal proteins, and unidentified retina proteins. Autoantibodies against CAII, GAPDH, aldolase, and α-enolase were tested in all 44 patients. Antibodies toward arrestin, tubulin, HSP27, and PKM2 were tested in 42 out of 44 patients, while antibodies against recoverin, HSP60, Rab6, and TULP1 were tested in 22 out of 44 patients. Antibodies against CRMP and the unidentified retinal proteins were tested in the first 20 patients. As illustrated in Fig. 2a,b, the antibody against α-enolase had the highest prevalence (65.9%) in the glycolytic enzyme-specific autoantibody panel, followed by antibodies against aldolase (40.9%), GAPDH (36.4%), and PKM2 (21.4%). In the panel of retinal proteins, anti-CAII showed the highest prevalence (43.2%), followed by antibodies against arrestin (27.3%) and tubulin (26.2%). The remaining antibodies in this panel, including antibodies against recoverin, HSP27, HSP60, CRMP, TULP1, and Rab6, contributed to a prevalence of less than 15% each. Except for anti-60 kDa (15%), all antibodies against the unidentified retinal proteins had a prevalence of no more than 10%.

Ten out of 12 patients with underlying autoimmune disease and both CAR patients had at least one autoantibody against the glycolytic enzymes. In patients without underlying autoimmune disease and malignancy, 27 out of 30 patients had at least one anti-glycolytic enzyme autoantibody. We found no significant difference between the two groups (*P* = 0.67, odds ratio 0.67) using Fisher exact test. Moreover, two CAR patients showed autoantibodies against recoverin, HSP60, and Rab6.

Out of 41 autoantibodies, 13 autoantibodies against the retinal proteins and glycolytic enzymes were analyzed further for the association with demographic (gender, age of onset, age at diagnosis, duration from onset to diagnosis), baseline (color vision, ERG, condition of the EZ, and occurrence of nyctalopia) and follow up variables (changes in BCVA, VF, OCT, and EZ). Aldolase was significantly associated with male gender in a Fisher exact test (*P* = 0.012, odds ratio 7.11, 95% CI 1.54–32.91). CAII showed significant association with age of onset (*P* = 0.025, 95% CI −17.28 to − 1.24) and PKM2 with age at diagnosis (*P* = 0.033, 95% CI 0.77–17.34) using independent t-test. We found significant association between disease duration from onset to diagnosis and anti-recoverin (*P* = 0.024), anti-CAII (*P* < 001), and anti-GAPDH (*P* = 0.045) by Mann–Whitney U test. Autoantibodies against the glycolytic enzymes, GAPDH (*P* = 0.001, odds ratio 1.87, 95% CI 1.32–2.64) and α-enolase (*P* = 0.002, odds ratio 4.37, 95% CI 1.83–10.37), were significantly associated with ERG findings using Fisher exact test. HSP27 was associated with BCVA in both eyes (right eye *P* = 0.001, 95% CI 0.26–0.88, left eye *P* < 0.001, 95%CI 0.38–1.01), while α-enolase, and PKM2 were associated with BCVA of the right eye at the last visit (*P* = 0.029, 95% CI − 0.97 to − 0.05, and *P* = 0.013, 95% CI 0.11–0.92, respectively) by independent t-test.

## Discussion

The data of autoantibodies in AIR patients from different populations is needed to validate the diagnostic criteria for this disease. In this study, we explored the autoantibody profile of 44 Thai patients with suspected AIR. With a female predominance and a median age of onset of 48 years, we found two CAR patients and 12 patients with underlying autoimmune diseases. Together with the clinical presentations and ophthalmological examination, we found 41 autoantibodies against retinal proteins, which were classified into glycolytic enzymes, retinal proteins, and other unidentified proteins.

The late age of onset is one of the distinctive characteristics differentiating AIR from other inherited retinal diseases^11,14^. The median age of onset of AIR patients was reported in the fifth or sixth decades of life^12^. Patients with family history of autoimmune disease tend to have earlier age at diagnosis^16,17^. In our study, the overall median age of onset was 48 years. The median age of onset of patients with underlying autoimmune disease was significantly lower than those without underlying disease (40, IQR 37–51.25 vs 50, IQR 45–60.5 years). Interestingly, we found three patients with very young age of onset (17–20 years), one of which had underlying autoimmune disease, without any history of inherited retinal diseases. Previous studies also reported occasional patients with a young age of onset (11–20 years)^16–18^. This should be a point of consideration for the clinicians to include AIR as a differential diagnosis of retinal diseases in young patients. For CAR patients, the age at diagnosis (62–79 years) are older than those with npAIR (36–55 years)^16,18^. Our CAR patients had the age at diagnosis of 58 and 63 years, which is comparable to the previous reports.

Underlying autoimmune diseases or family history of autoimmune disorders have the tendency to affect female^19^ and are often associated with npAIR^3,16^. The AIR patients in this cohort were predominantly female (33 in 44, 75%). Twelve out of 44 patients presented with underlying autoimmune disease, all of which were females. The most common underlying autoimmune disease in this study was SLE and RA, which were reported to primarily affect females^20^. SLE- and RA-related AIR has been reported occasionally in case reports^17,21,22^. Besides, hypothyroidism was also reported as the most common npAIR-related underlying autoimmune disease^17^.

In our study, the autoantibody against α-enolase, an important and ubiquitously expressed glycolytic enzyme, showed the highest prevalence, followed by anti-CAII autoantibody. Previous studies in AIR patients in the US and China reported similar trends of autoantibody profiles^2,23^. A study described the sensitivity and specificity of α-enolase as 92% and 31%, respectively, with a positive and negative predictive value of 43% and 87%, respectively^2^. The high prevalence of anti-α-enolase autoantibody might be induced by the molecular mimicry toward microbial and host enolases^24,25^. Another plausible explanation is the response against tumors since the highly mitotic cancer cells produce energy by aerobic glycolysis to promote tumor growth and metastasis^26–28^, which triggers the immune response to produce antibodies against the glycolytic enzymes^29,30^. The high rate of aerobic glycolysis occurring in the retinal cells, particularly the photoreceptors, renders the retina to be more sensitive to anti-glycolytic enzyme autoantibodies^31^. Anti-α-enolase autoantibody can penetrate the retinal tissue, reach its targets in the GCL and INL, and induce apoptotic cell death^32^. The autoantibody against CAII was also reported to induce target cell apoptosis by deranging the cells’ metabolism through the inhibition of this protein’s catalytic activities^33,34^. Anti-CAII is thus often associated with more severe clinical outcomes in AIR patients^34^, although we did not find significant association between anti-CAII autoantibody and the disease outcomes in this study.

In this female predominant disease, we found association between the third most common autoantibody in our patients, anti-aldolase and male gender. Risk estimate showed that anti-aldolase was seven times more likely to be found in male, but the significance of this finding requires further investigation.

Two of our patients had concurrent breast cancer and matched with the CAR criteria. The eye symptoms began two and seven years, respectively, before the diagnosis of cancer. Several types of cancers, including breast cancer, are reported to be associated with retinopathy^35^. The latency, or period from cancer diagnosis to onset of retinopathy or vice versa, was variable. A cohort study by Adamus et al. found that the time from cancer diagnosis to the onset of retinopathy was dependent on the cancer type and kinetics, immune response to cancer, and the cancer-induced autoimmune response toward the retina^12^. Nevertheless, the early diagnosis of CAR and appropriate management might improve the visual prognosis in these patients.

Both CAR patients in this study presented with autoantibodies against recoverin, HSP60, and Rab6. Reported to be related to various types of cancers, anti-Rab6 autoantibody could be found in both npAIR and pAIR patients^2,9^. The autoantibody against Rab6 is not detected in the normal population, thus making it a potentially specific biomarker for AIR, although the prevalence is low^10^. On the contrary, anti-HSP60 represents a natural antibody developed toward common pathogen invasions^36^ and the immunopathogenesis is hypothesized to occur through the molecular mimicry^37,38^. Autoantibody against HSP60 has also been related to several autoimmune diseases^37,39^, various types of cancer^2^, and even the normal population, although the concentration among these groups significantly differs^23^.

Autoantibody targeting recoverin, a photoreceptor-specific protein, has been postulated to be a good biomarker for AIR due to its absence in the normal population^2,6,9^. Moreover, it is expressed in several tumors, making it a sensitive and specific autoantibody for CAR^8^. In our study, anti-recoverin was found in three out of 22 tested patients, two of which were CAR patients and one with family history of malignancy.

Previous reports suggested a worse prognosis in patients with positive anti-α-enolase antibodies^40^. We found significant association between anti-GAPDH, anti-α-enolase and nonrecordable ERG, which represents the absence of electrical response in the retina indicating the severe dysfunction. Thirteen patients with positive anti-GAPDH who were tested for ERG showed nonrecordable waves. Of the 25 patients with positive anti-α-enolase and had the ERG reports, 23 of them showed nonrecordable ERG. Thus, our data not only confirmed the significance of anti-α-enolase antibody, but also provided additional information regarding autoantibody against GAPDH as a possible predictor for disease severity.

Anti-HSP27 autoantibody has been reported to cause symmetric, slow progression in CAR patients^9^. In our study, anti-HSP27 showed significant association with the BCVA at the last visit. All three patients with positive anti-HSP27 had good BCVA in both eyes at the time of diagnosis and the BCVA was stable or slightly decreased at the last follow up. However, the duration of follow up in these patients was less than 2 years. Thus, a higher number of patients is required to confirm the clinical correlation.

The primary therapy for AIR is a single or combination use of immunomodulatory agents, including corticosteroids, anti-metabolites, and T cell inhibitors^41^. A positive response to AIR treatment, as characterized by stable vision, improvement of VF and b-wave amplitude in ERG, and stable or even improvements of the EZ^16,42^, usually takes as long as one year or more before results could be observed^8,41^. In our study, 32 out of 44 patients received immunomodulators, where we found significant association between the treatment and the clinical outcomes of VF, OCT and condition of the EZ. Stable or improved BCVA and stable condition of EZ in one or both eyes were observed in 53% and 71% of patients receiving treatments, respectively. This positive response is in accordance with a previous study reporting 70% response rate after treatment with prednisone, cyclosporine, and azathioprine in a pool of CAR and npAIR patients^16^. We noticed that the therapeutic response in patients with underlying autoimmune disease was lower than those without (56% vs 77%). This finding corresponds to a study by Ferreyra et al. reporting the lesser response in patients with history of autoimmune diseases, suggesting that these patients might require stronger immunomodulatory treatments^16^.

Altogether, our study provided the correlation of autoantibody profiles and clinical presentations of AIR patients in Thailand. Since this study was not intended to identify antiretinal autoantibodies as the biomarkers for AIR, the autoantibody profile in the healthy population was beyond the scope of this study. In the future, we plan to assess the prevalence of autoantibody in healthy individuals and patients with other retinal dystrophies compared to AIR patients in the same population. This information could support the significance of these autoantibodies leading to rapid and proper management of AIR.

## Supplementary Information

Supplementary Tables.

## Data Availability

The datasets generated and/or analyzed during the current study are available from the corresponding author on reasonable request.
