# Supplementary material for: Autoantibody profiles and clinical association in Thai patients with autoimmune retinopathy
Source: Sci Rep. 2021 Jul 22;11:15047. doi: 10.1038/s41598-021-94377-0 (PMC8298708; doi:10.1038/s41598-021-94377-0)
Supplement: Supplementary file 1 — Supplementary Tables. [file 41598_2021_94377_MOESM1_ESM.docx]

**Autoantibody profiles and clinical association in Thai patients with autoimmune retinopathy**

Aulia Rahmi Pawestri^1§^, Niracha Arjkongharn^2§^, Ragkit Suvannaboon^2,3§^, Aekkachai Tuekprakhon^2,4^, Vichien Srimuninnimit^5^, Suthipol Udompunthurak^6^, La-ongsri Atchaneeyasakul^2^, Ajchara Koolvisoot^7*^, Adisak Trinavarat^2*^

^1^Faculty of Medicine, Universitas Brawijaya, Malang, Indonesia

^2^Department of Ophthalmology, Faculty of Medicine Siriraj Hospital, Mahidol University, Bangkok, Thailand

^3^Research Division, Faculty of Medicine Siriraj Hospital, Mahidol University, Bangkok, Thailand

^4^Nuffield Department of Medicine, Welcome center for Human Genetics, University of Oxford, Oxford, UK

^5^Division of Medical Oncology, Department of Medicine, Faculty of Medicine Siriraj Hospital, Mahidol University, Bangkok, Thailand

^6^Clinical Epidemiology Division, Siriraj Medical Research Center, Faculty of Medicine Siriraj Hospital, Mahidol University

^7^Division of Rheumatology, Department of Medicine, Faculty of Medicine Siriraj Hospital, Mahidol University, Bangkok, Thailand

^§^Authors contributed equally to this manuscript

***Co-corresponding authors**

Ajchara Koolvisoot, M.D., Division of Rheumatology, Department of Medicine, Faculty of Medicine Siriraj Hospital, 2 Wanglang Road, Bangkok Noi, Bangkok 10700, Thailand, +66 8 9897 6620, [ajchara.koo@mahidol.ac.th](mailto:ajchara.koo@mahidol.ac.th); and Adisak Trinavarat, M.D., Department of Ophthalmology, Faculty of Medicine Siriraj Hospital, 2 Wanglang Road, Bangkok Noi, Bangkok 10700, Thailand, +66 9 0990 5109, [adisak.tri@mahidol.ac.th](mailto:adisak.tri@mahidol.ac.th)

**Supplementary Table 1. Demographic and baseline data**

| **AIR ID** | **Age of onset**  **(year)** | **Gender** | **Eye** | **BCVA**  **(logMAR)** | **Visual field (degree)** | | | | **Color vision** | **ERG** | **Clinical manifestation** | **Underlying condition** | **History of treatment** |
| --- | --- | --- | --- | --- | --- | --- | --- | --- | --- | --- | --- | --- | --- |
|  |  |  |  |  | **S** | **N** | **I** | **T** |  |  |  |  |  |
| 1 | 62 | F | OD  OS | 0.24  0.12 | 10  10 | 35  35 | 45  35 | 50  12.5 | Normal | moderately decreased rod and cone function | Blurred vision, nyctalopia, peripheral vision loss, eye pain, dry eye | SLE, no RP, no malignancy | IOL OS  chloroquine |
| 2 | 62 | F | OD  OS | 1.64  0.82 | 10  15 | 10  15 | 12.5  15 | 50  15 | NE | NR | Blurred vision, decreased visual field | No autoimmune, no RP, no malignancy | none |
| 3 | 54 | F | OD  OS | 1.04  0.82 | N/A  45 | N/A  N/A | N/A  N/A | 50  N/A | Total color blindness | NR | Blurred vision, nyctalopia, peripheral vision loss | No autoimmune, no malignancy, family history of vision loss | none |
| 4 | 39 | F | OD  OS | 0.7  2.3 | 10  12.5 | 10  10 | 10  10 | 50  10 | Total color blindness | NR | Blurred vision, nyctalopia, photosensitivity | RA, no malignancy, family history of retinal degeneration | Chloroquine |
| 5 | 40 | F | OD  OS | 0  0 | 15  10 | 45  50 | 60  50 | 50  15 | Normal | mildly to moderately decreased rod and cone function | Nyctalopia, peripheral vision loss | RA, no RP, no malignancy | none |
| 6 | 66 | M | OD  OS | 0.1  0.12 | 40  17.5 | 60  60 | 60  60 | 50  65 | Normal | absent rod function, moderately decreased cone function | Blurred vision, ring scotoma | No autoimmune, no RP, no malignancy | IOL OD |
| 7 | 45 | M | OD  OS | 0.8  0.6 | 10  45 | 10  12.5 | 10  12.5 | 50  12.5 | NE | NR | Blurred vision, nyctalopia, peripheral vision loss | No autoimmune, No malignancy | IOL OD |
| 8 | 34 | F | OD  OS | 0.9  0.76 | 10  10 | 10  10 | 10  10 | 50  25 | Total color blindness | NR | Blurred vision, nyctalopia | RA, No malignancy | none |
| 9 | 72 | F | OD  OS | 0.2  0.3 | 17.5  30 | 15  15 | 15  15 | 50  17.5 | Normal | markedly decreased cone, moderately decreased rod function | Blurred vision, peripheral vision loss | No autoimmune, no RP, no malignancy | IOL OD OS |
| 10 | 42 | F | OD  OS | 0.12  0.12 | 45  15 | 60  55 | 55  55 | 50  80 | Normal | normal cone, markedly decreased rod function | Nyctalopia, ring scotoma | No autoimmune, no RP, no malignancy | none |
| 11 | 70 | M | OD  OS | 2.7  0 | N/A  15 | N/A  10 | N/A  10 | 50  10 | NE | NR | Blurred vision, nyctalopia, | No autoimmune, no malignancy, mother has macular degeneration | IOL OD OS |
| 12 | 20 | F | OD  OS | 0.08  0.08 | 10  15 | 40  35 | 35  35 | 50  50 | Normal | mildly decreased cone, absent rod function | Nyctalopia, peripheral vision loss | SLE, UCTD, no RP, no malignancy | none |
| 13 | 64 | F | OD  OS | 0.48  0.6 | 15  10 | 15  15 | 15  15 | 50  15 | NE | NR | Blurred vision, photoaversion, photosensitivity, hemeralopia | No autoimmune, granddaughter with retinoblastoma, No malignancy | none |
| 14 | 55 | F | OD  OS | 0.48  0.24 | 15  N/A | 30  30 | 25  30 | 50  45 | Normal | NR | Nyctalopia | No autoimmune, Glaucoma, no malignancy (history of malignancy in mother & aunt) | Laser treatment for diabetic retinopathy |
| 15 | 40 | F | OD  OS | 0  0 | 15  N/A | 15  55 | 60  55 | 50  15 | Normal | moderately decreased cone and rod function | Blurred vision, nyctalopia, peripheral vision loss, scotoma ring & paracentral | No autoimmune, no RP, no malignancy | none |
| 16 | 43 | F | OD  OS | 0.6  0.6 | 10  N/A | 30  10 | 45  10 | 50  10 | NE | NR | Blurred vision, nyctalopia, scotoma central & paracentral | SLE, no RP, no malignancy | IOL OD OS  chloroquine |
| 17 | 72 | M | OD  OS | 2.7  2.7 | N/A  20 | N/A  N/A | N/A  N/A | 50  N/A | NE | NR | Blurred vision | No autoimmune, no RP, no malignancy | IOL OD OS |
| 18 | 38 | F | OD  OS | 0.4  2.7 | 12.5  10 | 10  N/A | 10  N/A | 50  N/A | Normal | N/A | Blurred vision | Minimal change disease, family history of SLE (sister), abnormal mammogram, small nodule in left kidney (no proven malignancy) | IOL OS |
| 19 | 55 | F | OD  OS | 2.7  0.84 | N/A  30 | N/A  N/A | N/A  N/A | 50  N/A | NE | NR | Blurred vision | SLE, no RP, no malignancy | IOL OD OS  Chloroquine |
| 20 | 65 | F | OD  OS | 0.2  0.2 | 20  15 | 25  25 | 35  25 | 50  30 | Normal | moderately decreased cone and rod function | No symptoms | No autoimmune, diabetic retinopathy, no malignancy | none |
| 21 | 48 | F | OD  OS | 1.36  1.26 | 10  10 | 10  10 | 15  10 | 50  12.5 | NE | NR | Blurred vision, nyctalopia, peripheral vision loss | No autoimmune, no RP, breast cancer | IOL OS |
| 22 | 35 | F | OD  OS | 1.51  1.51 | 30  30 | 45  40 | 50  50 | 70  65 | Total color blindness | NR | Blurred vision, nyctalopia, central scotoma | No autoimmune, no RP, no malignancy | LASIK for myopia |
| 23 | 56 | F | OD  OS | 0.3  0.2 | 12.5  15 | 17.5  17.5 | 15  17.5 | 50  15 | Normal | NR | Nyctalopia | No autoimmune, no RP, no malignancy | none |
| 24 | 45 | M | OD  OS | 0.16  0.16 | 15  N/A | 15  15 | 15  15 | 50  15 | Normal | NR | Blurred vision, nyctalopia | No autoimmune, no RP, no malignancy | none |
| 25 | 52 | M | OD  OS | 0.24  1.64 | 40  12.5 | 55 | 50  60 | 50  65 | Total color blindness | moderately decreased cone and rod function | Blurred vision, nyctalopia, scotoma ring | No autoimmune, retinal degeneration, no malignancy | IOL OD OS |
| 26 | 45 | M | OD  OS | Prosthesis  0 | N/A  12.5 | N/A  45 | N/A  45 | 50  30 | N/A | N/A | Nyctalopia | No autoimmune, no RP, no malignancy | Enucleation OD (prosthesis) |
| 27 | 50 | F | OD  OS | 2.3  2.3 | 10  15 | 10  N/A | 10  N/A | 50  N/A | NE | NR | Blurred vision, nyctalopia, peripheral vision loss | SLE, no RP, no malignancy | IOL, OD OS |
| 28 | 55 | M | OD  OS | 0.02  0.02 | 12.5  45 | 12.5  12.5 | 10  12.5 | 50  12.5 | Normal | NR | Nyctalopia, peripheral vision loss | No autoimmune, no RP, no malignancy | IOL, OD OS |
| 29 | 62 | M | OD  OS | 0.52  0 | 12.5  50 | 15  15 | 15  15 | 50  15 | Normal | NR | Blurred vision, nyctalopia, peripheral vision loss | No autoimmune, no RP, no malignancy  Vit D insufficiency | IOL, OD |
| 30 | 74 | F | OD  OS | 0.14  0.1 | 15  10 | 17.5  20 | 15  20 | 50  15 | Normal | NR | Blurred vision, nyctalopia | SLE, autoimmune cytopenia, no malignancy | IOL, OD OS |
| 31 | 28 | F | OD  OS | 0.1  0.1 | 40  12.5 | 20  25 | 40  25 | 50  50 | Normal | NR | Nyctalopia, peripheral vision loss, scotoma paracentral | No autoimmune, family history of SLE (aunt), no RP, no malignancy | none |
| 32 | 48 | F | OD  OS | 0.32  0.32 | 10  10 | 12.5  15 | 10  15 | 50  10 | Normal | NR | Nyctalopia, peripheral vision loss | No autoimmune, mother had unknown cause of peripheral vision loss, no malignancy | none |
| 33 | 60 | F | OD  OS | 0.3  1.02 | 15  40 | 15  15 | 15  15 | 50  12.5 | Normal | NR | Peripheral vision loss | No autoimmune, grandmother had unknown cause of vision loss, no malignancy | IOL, OD OS |
| 34 | 48 | M | OD  OS | 2.3  2.3 | 10  10 | 15  N/A | 20  N/A | 50  N/A | N/A | NR | Blurred vision | No autoimmune, central retinal vein occlusion, no malignancy | IOL, OD OS |
| 35 | 40 | F | OD  OS | 0.5  0.56 | 10  15 | 10  10 | 10  10 | 50  12.5 | Total color blindness | NR | Nyctalopia | RA, no malignancy | Chloroquine |
| 36 | 50 | F | OD  OS | 0  0.04 | 35  45 | 45  50 | 60  50 | 50  45 | Normal | markedly decreased rod and cone function | Photopsia, scotoma paracentral | No autoimmune, no RP, no malignancy | none |
| 37 | 17 | M | OD  OS | 0  1.04 | 45  N/A | 45  55 | 60  55 | 50  70 | Normal | decreased amplitude of cone function, normal mesopic | No obvious symptoms | No autoimmune, no RP, no malignancy | None |
| 38 | 55 | F | OD  OS | 0.2  0.14 | 30  30 | 40  35 | 40  35 | 45  30 | Partial color blindness | NR | Nyctalopia | No autoimmune, no RP, no malignancy | none |
| 39 | 48 | F | OD  OS | 0.1  0.1 | 25  15 | 60  15 | 65  15 | 60  15 | Normal | moderately decreased cone and rod function OD, NR OS | Peripheral vision loss, photopsia | No autoimmune, no RP, no malignancy | none |
| 40 | 50 | F | OD  OS | 0.3  0.3 | 10  10 | 10  10 | 17  15 | 10  10 | Normal | NR | Nyctalopia, peripheral vision loss | No autoimmune, no RP, breast cancer | none |
| 41 | 46 | F | OD  OS | 0.1  0.1 | 10  10 | 15  15 | 15  15 | 15  15 | Normal | NR | Nyctalopia, peripheral vision loss | No autoimmune, no RP, no malignancy | none |
| 42 | 44 | F | OD  OS | 0.1  0.1 | 12  15 | 18  15 | 12  14 | 25  15 | Normal | NR | peripheral vision loss | No autoimmune, no RP, no malignancy | none |
| 43 | 20 | F | OD  OS | 0.0  0.0 | 40  40 | 12  50 | 60  50 | 50  15 | Normal | NR | Nyctalopia, paracentral scotoma | Pemphigus vulgaris, no RP, no malignancy | none |
| 44 | 38 | F | OD  OS | 0.1  0.0 | N/A^c^  N/A^c^ | N/A^c^  N/A^c^ | N/A^c^  N/A^c^ | N/A^c^  N/A^c^ | Normal | NR | shadow obscured temporal VF OD | No autoimmune, retinal vasculitis, no malignancy | Laser for retinal vasculitis |

BCVA: best corrected visual acuity, ERG: electroretinogram, F: female, M: male, m: month, y: year, logMAR: logarithm of minimum angle of resolution, IOL: intraocular lens, OD: oculus dexter, OS: oculus sinister, RA: rheumatoid arthritis, SLE: systemic lupus erythematosus, UCTD: undifferentiated connective tissue disease.

S: superior, N: nasal, I: inferior, T: temporal, N/A: not applicable, NE: cannot be evaluated due to low fixation, NR: nonrecordable

^c^ VF was evaluated using automated perimetry and is thus unable to be compared.

**Supplementary Table 2. Disease progression of patients with autoimmune retinopathy**

| **AIR ID** | **Eye** | **BCVA**  **(logMAR)** | | **Visual field** | | **OCT** | | **Duration of follow up**  **(year)** | **IHC** | **Anti-retinal antibody** | **Treatment** | |
| --- | --- | --- | --- | --- | --- | --- | --- | --- | --- | --- | --- | --- |
|  |  | **Baseline** | **Last visit** | **Findings** | **Duration between VF evaluation**  **(year)** | **Findings** | **Duration between OCT examination (year)** |  |  |  | **Regiment** | **Duration from onset until first treatment**  **(year)** |
| 1 | OD  OS | 0.24  0.12 | 0.12  0.12 | Decreased  Decreased | 6.8 | Worsen with foveal preservation | 5 | 6 | N/A | Aldolase | Prednisolone, methotrexate | 3 |
| 2 | OD  OS | 1.64  0.82 | 2.3  1.3 | Stable  Decreased | 2.4 | Stable | 2 | 2.5 | Positive | Enolase, CRMP, 44-kDa | Prednisolone, mycophenolate | 5 |
| 3 | OD  OS | 1.04  0.82 | 2.7  2.7 | Decreased  Decreased | 2.6 | Worsen severely | 7 | 6.25 | Positive | CAII, HSP27, aldolase, enolase, tubulin, PKM2 | Prednisolone, dexamethasone, cyclosporine, mycophenolate, IVIG, rituximab | 2 |
| 4 | OD  OS | 0.7  2.3 | 0.4  1.47 | Stable  Stable | 3.1 | Worsen severely | 6 | 3.8 | Positive | CAII, enolase | Prednisolone, methotrexate | 11 |
| 5 | OD  OS | 0  0 | 0.2  0 | Decreased  Decreased | 1.5 | Worsen mildly | 0.5 | 0.5 | Positive | Aldolase, arrestin | Prednisolone, azathioprine | 10 |
| 6 | OD  OS | 0.1  0.12 | 0.18  0.14 | Decreased  Decreased | 5.3 | Lesion persists in ONL | 5 | 4.7 | Positive | CAII, 32 kDa, aldolase | Prednisolone, cyclosporin | 0.25 |
| 7 | OD  OS | 0.8  0.6 | 1.44  0.56 | Stable  Decreased | 4.7 | Stable (bad) | 5 | 4.2 | Positive | GAPDH, aldolase, enolase, CRMP | Prednisolone, methotrexate, mycophenolate | 7 |
| 8 | OD  OS | 0.9  0.76 | 1.9  2.3 | Stable  Stable | 5.4 | Worsen | 10 | 5 | Positive | 33 kDa, 92 kDa, 121 kDa | Prednisolone, methotrexate | 17 |
| 9 | OD  OS | 0.2  0.3 | 0.1  0.1 | Stable  Stable | 0.7 | Normal |  | 0.7 | Positive | GAPDH, tubulin, 27 kDa, 44 kDa, | Prednisolone | 1 |
| 10 | OD  OS | 0.12  0.12 | 0.1  0.08 | Decreased  Decreased | 1.7 | Stable | 2 | 2 | Positive | GAPDH, enolase, 136 kDa | Prednisolone, azathioprine | 2 |
| 11 | OD  OS | 2.7  0 | 2.9  0.12 | NE  Stable | 3.8 | Worsen mildly | 5 | 4.3 | Positive | CAII, enolase | Prednisolone, methotrexate | 2 |
| 12 | OD  OS | 0.08  0.08 | 0.1  2.3 | Improved  Decreased | 4.7 | Stable (bad) | 5 | 4.2 | Negative | Enolase  23 kDa, | Prednisolone, azathioprine | 30 |
| 13 | OD  OS | 0.48  0.6 | 0.7  0.42 | Decreased  Decreased | 1.8 | Stable | 2 | 1.75 | Negative | CAII, aldolase, arrestin, 24 kDa, 47 kDa, | N/A | - |
| 14 | OD  OS | 0.48  0.24 | 0.74  0.56 | Decreased  Decreased | 3.8 | PDR+ERM, VMT | 4 | 5.7 | Negative | CAII, 35 kDa, 36 kDa | Prednisolone, azathioprine | 12 |
| 15 | OD  OS | 0  0 | 0.14  0.06 | Decreased  Decreased | 4.8 | Stable (normal) | 5 | 3.8 | Negative | 22 kDa, 135 kDa, 220 kDa | Prednisolone, azathioprine | 11 |
| 16 | OD  OS | 0.6  0.6 | 1.58  1.85 | Decreased  Decreased | 3.8 | Worsen mildly | 4 | 6 | Positive | Enolase, 60 kDa, 70 kDa, 72 kDa | Prednisolone, azathioprine | 21 |
| 17 | OD  OS | 2.7  2.7 | 2.7  2.7 | NE  NE | - | Worsen | 2 | 2.6 | Negative | CAII, arrestin, GAPDH, enolase, tubulin, 28 kDa, 60 kDa, 76 kDa, 82 kDa, 96 kDa, 112 kDa | Prednisolone, mycophenolate | 3 |
| 18 | OD  OS | 0.4  2.7 | 0.44  2.7 | Stable  NE | 3.6 | Worsen | 7 | 1 | Positive | 23 kDa, 70 kDa, 102 kDa | N/A | - |
| 19 | OD  OS | 2.7  0.84 | 2.9  2.3 | NE  NE | - | N/A |  | 1 | Positive | CAII, enolase, 42 kDa, 60 kDa, 82 kDa, 136 kDa | N/A | - |
| 20 | OD  OS | 0.2  0.2 | 0.24  0.2 | Improved  Improved | 2.5 | Normal | 3 | 2.7 | Positive | CAII, 32 kDa, 40 kDa | N/A | - |
| 21 | OD  OS | 1.36  1.26 | 1.36  1.06 | Stable  Stable | 2.5 | Worsen mildly | 2 | 2 | Positive | Recoverin, GAPDH, Rab6, enolase, HSP60, TULP1 | Prednisolone, mycophenolate | 10 |
| 22 | OD  OS | 1.51  1.51 | 1.34  1.44 | Decreased  Decreased | 1.6 | Stable | 1 | 1.5 | N/A | Aldolase, enolase, arrestin | Prednisolone, mycophenolate | 16 |
| 23 | OD  OS | 0.3  0.2 | 0.1  0.1 | Decreased  Stable | 2.7 | Stable | 3 | 1.7 | N/A | CAII | N/A | - |
| 24 | OD  OS | 0.16  0.16 | 0.1  0.22 | N/A  N/A | - | N/A |  | 0.5 | N/A | Aldolase, enolase, arrestin, tubulin | N/A | - |
| 25 | OD  OS | 0.24  1.64 | 0.3  2 | N/A  N/A | - | N/A |  | 0.25 | N/A | Aldolase, arrestin, tubulin, PKM2 | N/A | - |
| 26 | OD  OS | Prosthesis  0 | Prosthesis  0 | NE  Decreased | 1.3 | Normal | 3 | 2.75 | N/A | GAPDH, aldolase, enolase, tubulin, PKM2 | Dexamethasone, azathioprine | 7 |
| 27 | OD  OS | 2.3  2.3 | 1.56  2.3 | Stable  NE | 1.8 | Stable | 3 | 2.6 | N/A | Aldolase, enolase | Prednisolone, mycophenolate | 12 |
| 28 | OD  OS | 0.02  0.02 | 0.04  0 | Decreased  Decreased | 1.2 | Stable | 1 | 1.6 | N/A | CAII, GAPDH. enolase, arrestin | Prednisolone, azathioprine | 6 |
| 29 | OD  OS | 0.52  0 | 0.3  0 | Decreased  Stable | 0.9 | Worsen mildly | 1 | 1 | N/A | CAII, GAPDH, aldolase, enolase | Prednisolone, mycophenolate | 2 |
| 30 | OD  OS | 0.14  0.1 | 0.1  0.2 | N/A  N/A | - | Normal |  | 12.25 | N/A | CAII, GAPDH, aldolase, arrestin | N/A | - |
| 31 | OD  OS | 0.1  0.1 | 0.1  0.1 | Decreased  Decreased | 1.8 | Stable | 2 | 1.25 | N/A | GAPDH, enolase, PKM2 | Prednisolone, methotrexate | 2 |
| 32 | OD  OS | 0.32  0.32 | 0.2  0.2 | N/A  N/A | - | N/A |  | 0.25 | N/A | CAII, GAPDH, arrestin, tubulin, PKM2 | N/A | - |
| 33 | OD  OS | 0.3  1.02 | 0.16  1.1 | Improved  Improved | 0.7 | Stable | 1 | 0.25 | N/A | GAPDH, aldolase, enolase, tubulin | Prednisolone, azathioprine | 16 |
| 34 | OD  OS | 2.3  2.3 | 1  2.3 | N/A  N/A | - | Stable | 1 | 0.4 | N/A | Aldolase, enolase, PKM2 | Prednisolone, methotrexate | 15 |
| 35 | OD  OS | 0.5  0.56 | 0.6  0.6 | Stable  Stable | 0.7 | Stable | 1 | 0.3 | N/A | Enolase | Prednisolone, methotrexate, azathioprine, leflunomide | 25 |
| 36 | OD  OS | 0  0.04 | 0.02  0.1 | Decreased  Decreased | 0.7 | N/A |  | 0.008 | N/A | PKM2 | Prednisolone, azathioprine | 7 |
| 37 | OD  OS | 0  1.04 | 0  1.04 | N/A  N/A | - | Normal |  | 1.75 | N/A | Aldolase, enolase | Prednisolone | 7 |
| 38 | OD  OS | 0.2  0.14 | 0.14  0.14 | Decreased  Decreased | 5.7 | Thin retina |  | 6 | N/A | GAPDH, aldolase, enolase, arrestin | N/A | - |
| 39 | OD  OS | 0.1  0.1 | 0.1  0.12 | N/A  N/A | - | OD normal OS abnormal |  | 0.17 | N/A | CAII, enolase, tubulin. Hsp27 | N/A | - |
| 40 | OD  OS | 0.3  0.3 | 0.1  0.1 | Decreased  Decreased | 1.5 | Stable | 1 | 1.5 | Positive | Recoverin, Rab6, enolase, HSP60 | Prednisolone, azathioprine | 13 |
| 41 | OD  OS | 0.1  0.1 | 0.2  0.2 | Improved  Improved | 1.8 | Stable | 1 | 1.8 | N/A | CAII, GAPDH, HSP27, enolase, tubulin, PKM2 | Prednisolone, mycophenolate, azathioprine | 2 |
| 42 | OD  OS | 0.1  0.1 | 0.0  0.0 | N/A  N/A | - | N/A |  | 1 | N/A | CAII, GAPDH, enolase, arrestin | N/A | - |
| 43 | OD  OS | 0.0  0.0 | 0.2  0.2 | Decreased  Decreased | 1.9 | Stable | 2 | 2 | N/A | CAII, GAPDH, enolase, PKM2 | Prednisolone, azathioprine | 23 |
| 44 | OD  OS | 0.1  0.0 | 0.2  0.2 | N/A  N/A | - | Stable | 1 | 1 | N/A | CAII, HSP27, aldolase, enolase, tubulin, PKM2 | Prednisolone | 3 |

BCVA: best corrected visual acuity, IHC: immunohistochemistry, OCT: optical coherence tomography, OD: oculus dexter, OS: oculus sinister, logMAR: logarithm of minimum angle of resolution VF: visual field, N/A: not applicable

CAII: carbonic anhydrase II, CRMP: collapsin response mediator protein, GAPDH: glyceraldehyde 3-phosphate dehydrogenase, Hsp: heat-shock protein, PKM2: pyruvate kinase isozyme M2, TULP1: tubby-like protein 1, kDa: kilo Dalton
